# Ultrastructure of Zika virus particles in cell cultures

**DOI:** 10.1590/0074-02760160104

**Published:** 2016-07-11

**Authors:** Debora Ferreira Barreto-Vieira, Ortrud Monika Barth, Marcos Alexandre Nunes da Silva, Carolina Cardoso Santos, Aline da Silva Santos, Joaquim Batista F, Ana Maria Bispo de Filippis

**Affiliations:** 1 Fundação Oswaldo Cruz, Instituto Oswaldo Cruz, Laboratório de Morfologia e Morfogênese Viral, Rio de Janeiro, RJ, Brasil; 2 Fundação Oswaldo Cruz, Instituto Oswaldo Cruz, Laboratório de Flavivírus, Rio de Janeiro, RJ, Brasil; 3 Laboratório Central de Saúde Pública do Espírito Santo/Virologia, Vitória, ES, Brasil

**Keywords:** Zika virus, virus particles, cell culture, transmission electron microscopy

## Abstract

Zika virus (ZIKV) has infected thousands of Brazilian people and spread to other American countries since 2015. The introduction of ZIKV brought a strong impact to public health in Brazil. It is of utmost importance to identify a susceptible cell line that will enable the isolation and identification of the virus from patient samples, viral mass production, and testing of drug and vaccine candidates. Besides real-time reverse transcriptase polymerase chain reaction diagnosis for detecting the viral genome, virus isolation in cell lines was useful in order to study the structure of the viral particle and its behaviour inside cells. Analysis of ZIKV infected cell lines was achieved using transmission electron microscopy (TEM). Blood was obtained from a Brazilian patient during the first days after presenting with signs of the disease, and ZIKV from the patient’s blood was isolated in the C6/36 mosquito cell line. Afterwards, Vero cells were inoculated with the viral suspension, fixed six days after inoculation, embedded in polymers, and ultra-thin cut. Like dengue viruses, this flavivirus showed numerous virus particles present inside cellular vesicles thereby confirming the susceptibility of the Vero cell line to ZIKV replication. TEM is a unique technique available to make the virus visible.

Zika virus (ZIKV), so-called because it was originally isolated from a rhesus monkey in the Zika Forest, Uganda, in 1947 (Dick et al. 1952), is a flavivirus transmitted to people mainly through the bite of an infected *Aedes* sp. mosquito (*A. aegypti* or *A. albopictus*). *Aedes* spp. mosquitoes that transmit also dengue, chikungunya, and yellow fever occur worldwide, and constitute a high risk for ZIKV global transmission. ZIKV infection is usually asymptomatic or causes mild symptoms, such as fever, rash, muscle/joint pain and conjunctivitis. Severe disease and fatalities are uncommon (Lucey & Gostin 2016). Infections in humans have occurred in several African and Asian countries. In 2007, an outbreak of ZIKV on Yap Island in the southwestern Pacific Ocean started as a relatively mild disease characterised by rash, arthralgia and conjunctivitis. This was the first time that ZIKV was detected outside of Africa or Asia (Duffy et al. 2009, Hayes 2009). In October 2013, French Polynesia recorded a large outbreak with a great number of cases, some of which presented neurological and autoimmune complications (Guillain-Barre syndrome). The clinical presentation is defined as a “dengue-like syndrome” (Loos et al. 2014).

In early 2015, ZIKV was detected by reverse transcriptase-polymerase chain reaction (RT-PCR) in the sera of eight patients from the Brazilian northeastern region, who presented symptoms of mild fever, rash, conjunctivitis and arthralgia (Zanluca et al. 2015). In addition, other symptoms have been observed that include microcephaly in newborns apparently resulting from ZIKV infection of the mothers during pregnancy (Higgs 2016). ZIKV currently circulates in 21 Brazilian states and is estimated to have infected between 440,000 to 1.3 million people in 2015. As of May 7, 2016, 7438 cases of microcephaly have been reported according to the surveillance protocol settings (newborn, stillbirth, abortion, or fetus). Of these suspected cases, 4004 cases were investigated and classified, whereas 3433 (46.2%) remain under investigation. Of the classified cases, 1326 were confirmed for microcephaly and/or central nervous system abnormalities suggestive of congenital infection and 2679 were discarded (TGHN 2015, MS 2016). Studies performed by Slovenian researchers (Mlakar et al. 2016) have detected ZIKV in microcephalic foetal brain tissue by real time RT-PCR. This finding was also consistent with electron microscopy observations. Furthermore, the complete genome of ZIKV was recovered from the foetal brain. The expectant mother had a febrile illness with rash at the end of the first trimester of pregnancy while she was living in Brazil. Since Brazil reported ZIKV in May 2015, infections have occurred in at least 20 countries, mainly in South and Central America. The Pan American Health Organization issued a series of epidemiological updates and alerts in 2015 urging for enhanced surveillance of ZIKV as well as for neurological, autoimmune and congenital malformation associations (PAHO/WHO 2015, Lucey & Gostin 2016).

Monolayers of Vero cells were inoculated with a blood sample from a ZIKV positive patient and analysed for the presence of ZIKV particles by transmission electron microscopy (TEM). The supernatants of the infected cells were tested by real time RT-PCR for the presence of ZIKV genomes.

The blood sample used was obtained from a patient residing in Vitória, Espiríto Santo, Brazil, in July 2015 and who presented with fever, myalgia, arthralgia, nausea, pruriginous exanthema as well as joint pain in the hands and feet. ZIKV was first isolated from the patient’s blood sample in the *A. albopictus* C6/36 cell line and then propagated in Vero cells. Vero cells were inoculated with 200 mL of C6/36 fluid that was adsorbed onto the cells for 1 h at 37ºC. After the incubation period, Minimum Essential Medium Eagle (MEM) supplemented with 2% foetal bovine serum was added and the cells were incubated at 37ºC. Six days after inoculation, the cell culture fluid was used for molecular analysis and the cell monolayer was processed for morphological analysis.

*Molecular analysis* - Vero cell culture fluid was subjected to quantitative ZIKV-specific real time RT-PCR (Lanciotti et al. 2008 *).* Viral RNA was extracted from 140 μL of the culture fluid using the QIAamp Viral RNA Mini Kit (QIAGEN, Valencia, CA, USA) in accordance with the manufacturer’s suggested protocol.

*Morphological analysis* - Cells were fixed with 1% glutaraldehyde in sodium cacodylate buffer (0.2 M, pH 7.2), post-fixed with 1% buffered osmium tetroxide, dehydrated in acetone, embedded in epoxy resin, and polymerised at 60ºC for three days (Sesso 2007, Barreto-Vieira et al. 2010, 2015). The resin blocks were then cut into 50-70 nm thick ultrathin sections. The sections were picked up on copper grids, stained with uranyl acetate and lead citrate, and observed in a Zeiss EM-900 TEM.

Ultrastructural analysis by transmission electron microscopy showed injury of Vero cell monolayers six days post-inoculation with ZIKV. Numerous myelin figures, vacuoles and mitochondria filled the cytoplasm. Viral particles of approximately 50 nm in diameter occurred as clusters inside vesicles (Fig. 1A-B). Viral nucleocapsids were also observed (Fig. 1C). Furthermore, viral RNA in the supernatants from the inoculated cell monolayers was detected by real time RT-PCR, confirming the presence of ZIKV.

**Figure:**
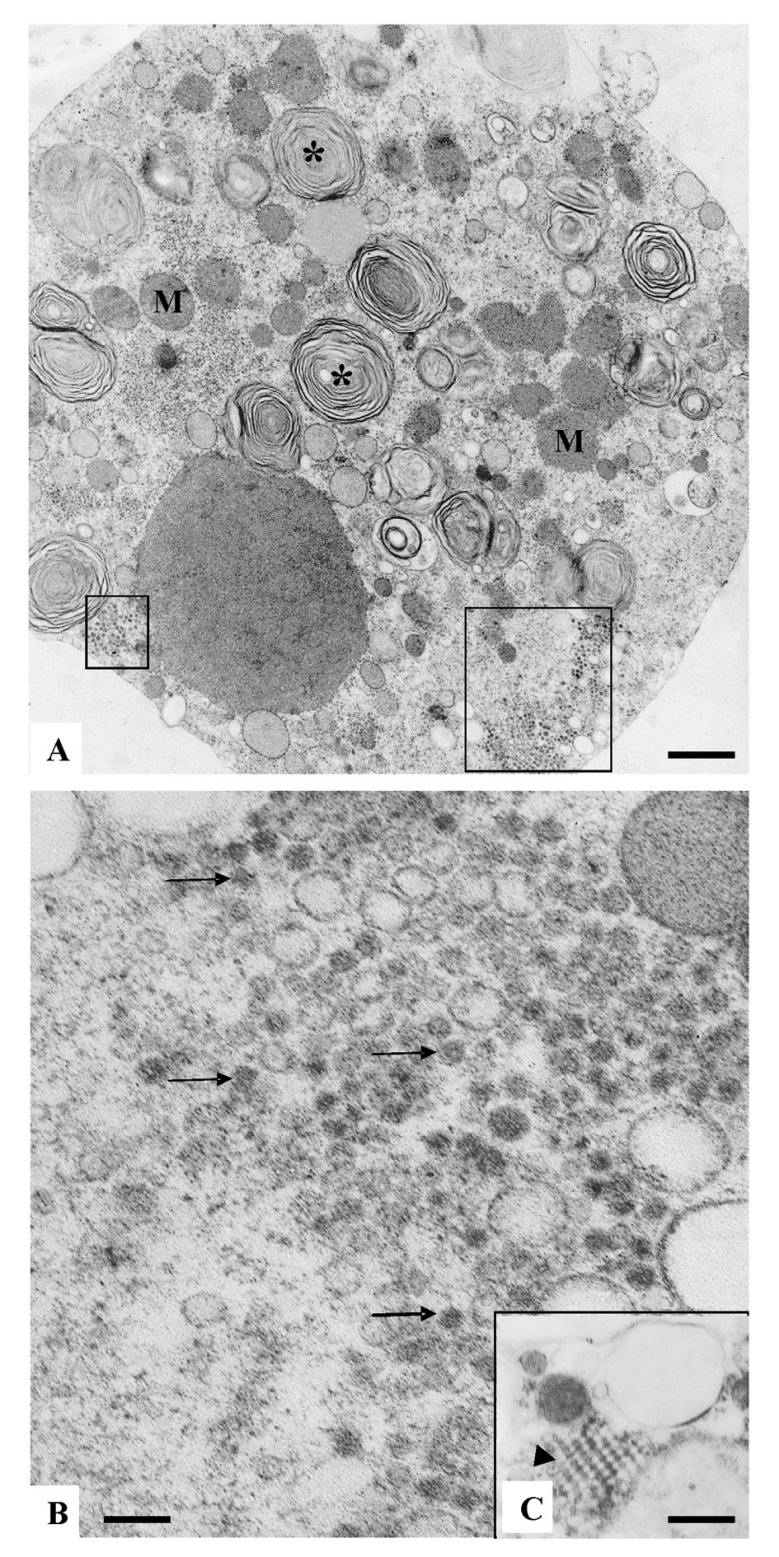
Vero cells six days post inoculation with a human blood serum sample positive for ZIKV. (A) Infected Vero cell presenting numerous myelin figures (*), vacuoles, and clusters of ZIKV particles (marked areas); bar = 700 nm; (B) ZIKV particles (arrows); bar = 120 nm; (C) regularly arranged viral nucleocapsids (arrow head); bar = 300 nm.

Vero cells are widely used in virology studies for their efficiency in replicating several viruses such as measles, poxviruses, dengue and rubeola (Takata et al. 1994, Barth 2000, Schatzmayr et al. 2009). In the present study, Vero cell cultures that were inoculated with a ZIKV positive human blood sample showed clusters of viral particles inside cytoplasmic vesicles. The diameter of these virus particles averaged 50 nm. Besides enveloped virus particles, nucleocapsids were also observed indicating that viral replication occurred. Replication of the virus was further confirmed by real time RT-PCR. These aspects confer with the morphological studies of dengue virus replication in C6/36 and Vero cells (Barth 2000).

The presence of ZIKV genomes in cultures of Vero cells was also confirmed by Calvet et al. (2016) using real time RT-PCR, thus corroborating ultrastructural observations made by transmission electron microscopy.

ZIKV proteins were detected by immunohistochemistry in human placental tissue (de Noronha et al. 2016). ZIKV particles were observed in cells from microcephalic foetal brain tissue (Mlakar et al. 2016) that were formerly detected by real time RT-PCR. The viral particles showed typical flavivirus features and were 50-57 nm in diameter. In addition, the cryo-electron microscopy structure of ZIKV particles presented a 50 nm diameter (Kostyuchenko et al. 2016, Sirohi et al. 2016). Experimentally infected human neural stem cells with ZIKV showed a typical flavivirus configuration (Garcez et al. 2016).

To date, the data presented on the morphological features of ZIKV particles in tissues and cell cultures are consistent.

## References

[B1] Barreto-Vieira DF, Barth-Schatzmayr OM, Schatzmayr HG (2010). Modelo animal experimental para o estudo da patogênese dos vírus dengue sorotipos 1 e 2. Manual de técnicas.

[B2] Barreto-Vieira DF, Takiya CM, Jácome FC, Rasinhas AC, Barth OM (2015). Secondary infection with dengue viruses in a murine model: morphological analysis. Indian J Appl Res.

[B3] Barth OM (2000). Atlas of dengue viruses morphology and morphogenesis.

[B4] Calvet G, Aguiar RS, Melo ASO, Sampaio SA, Filippis I, Fabri A (2016). Detection and sequencing of Zika virus from amniotic fluid of fetuses with microcephaly in Brazil: a case study. Lancet Infect Dis.

[B5] Noronha L, Zanluca C, Azevedo MLV, Luz KG, Santos CND (2016). Zika virus damages the human placental barrier and presents marked fetal neurotropism. Mem Inst Oswaldo Cruz.

[B6] Dick GW, Kitchen SF, Haddow AJ (1952). Zika virus. I. Isolation and serological specificity. Trans R Soc Trop Med Hyg.

[B7] Duffy MR, Chen TH, Hancock WT, Powers AM, Kool JL, Lanciotti RS (2009). Zika virus outbreak on Yap Island, Federated States of Micronesia. N Engl J Med.

[B8] Garcez PP, Loiola EC, Costa RM, Higa LM, Trindade P, Delvecchio R (2016). Zika virus impairs growth in human neurospheres and brain organoids. Science.

[B9] Hayes EB (2009). Zika virus outside Africa. Emerg Infect Dis.

[B10] Higgs S (2016). Zika virus: emergence and emergency. Vector-Borne Zoonotic Dis.

[B11] Kostyuchenko VA, Lim EXY, Zhang S, Fibriansah G, Ng TS, Ooi JS (2016). Structure of the thermally stable Zika virus. Nature.

[B12] Lanciotti RS, Kosoy OL, Laven JJ, Velez JO, Lambert AJ, Johnson AJ (2008). Genetic and serologic properties of Zika virus associated with an epidemic, Yap State, Micronesia, 2007. Emerg Infect Dis.

[B13] Loos S, Mallet HP, Goffart IL, Gauthier V, Cardoso T, Herida M (2014). Current Zika virus epidemiology and recent epidemics. Med Mal Infect.

[B14] Lucey DR, Gostin LO (2016). The emerging Zika pandemic: enhancing preparedness. JAMA.

[B15] Mlakar J, Korva M, Tul N, Popović M, Poljšak-Prijatelj M, Mraz J (2016). Zika virus associated with microcephaly. N Engl J Med.

[B16] MS - Ministério da Saúde (2016). Dengue, chikungunya e Zika. Informe epidemiológico de casos de microcefalia.

[B17] PAHO/WHO - Pan American Health Organization/World Health Organization (2015). Epidemiological alert: increase of microcephaly in the northeast of Brazil.

[B18] Schatzmayr HG, Simonetti BR, Abreu DC, Simonetti JP, Simonetti SR, Costa RVC (2009). Animal infections by vaccinia-like viruses in the state of Rio de Janeiro: an expanding disease. Braz J Vet Res.

[B19] Sesso A (2007). Fixação de sistemas biológicos. In: de Souza W, org. Manual sobre técnicas básicas em microscopia eletrônica.

[B20] Sirohi D, Chen Z, Sun L, Klose T, Pierson TC, Rossmann MG (2016). The 3.8 a resolution cryo-EM structure of Zika virus. Science.

[B21] Takata CS, Kubrusly FS, Miyaki C, Mendes IF, Rizzo E (1994). Susceptibility of Vero cell line to vaccine of the measles virus. Rev Saude Publica.

[B22] TGHN - The Global Health Network (2015). Zika infection.

[B23] Zanluca C, Melo VC, Mosimann AL, Santos GI, Santos CN, Luz K (2015). First report of autochthonous transmission of Zika in Brazil. Mem Inst Oswaldo Cruz.

